# Supplementary figures and images for: Characterization of transgenic mouse lines for selectively targeting satellite glial cells and macrophages in dorsal root ganglia
Source: PLoS One. 2020 Sep 11;15(9):e0229475. doi: 10.1371/journal.pone.0229475 (PMC7485865; doi:10.1371/journal.pone.0229475)

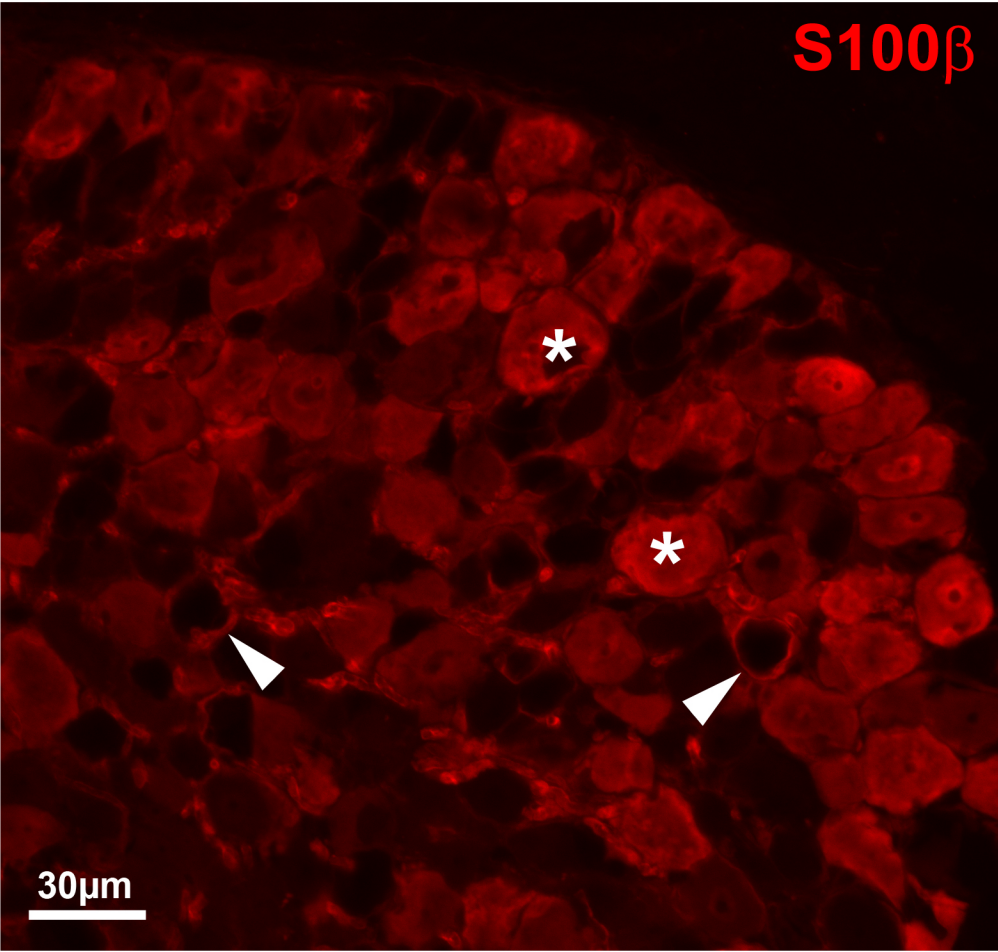

Supplement: S1 Fig — (PDF) [file pone.0229475.s001.pdf]
